# Evi1 defines leukemia-initiating capacity and tyrosine kinase inhibitor resistance in chronic myeloid leukemia

**DOI:** 10.1038/onc.2014.108

**Published:** 2014-04-21

**Authors:** T Sato, S Goyama, K Kataoka, R Nasu, T Tsuruta-Kishino, Y Kagoya, A Nukina, K Kumagai, N Kubota, M Nakagawa, S Arai, A Yoshimi, H Honda, T Kadowaki, M Kurokawa

**Affiliations:** 1 Department of Hematology and Oncology, Graduate School of Medicine, The University of Tokyo, Tokyo, Japan; 2 Department of Transfusion Medicine, The University of Tokyo Hospital, Tokyo, Japan; 3 Department of Metabolic and Diabetic Diseases, Graduate School of Medicine, The University of Tokyo, Tokyo, Japan; 4 Department of Disease Model, Research Institute for Radiation Biology and Medicine, Hiroshima University, Hiroshima, Japan

## Abstract

Relapse of chronic myeloid leukemia (CML) is triggered by stem cells with a reconstituting capacity similar to that of hematopoietic stem cells (HSCs) and CML stem cells are a source of resistance in drug therapy with tyrosine kinase inhibitors (TKIs). Ecotropic viral integration site 1 (EVI1), a key transcription factor in HSC regulation, is known to predict poor outcomes in myeloid malignancies, however, incapability of prospective isolation of EVI1-high leukemic cells precludes the functional evaluation of intraindividual EVI1-high cells. Introduction of CML into Evi1-internal ribosomal entry site (IRES)-green fluorescent protein (GFP) knock-in mice, a versatile HSC-reporter strain, enables us to separate Evi1-high CML cells from the individual. Evi1-IRES-GFP allele models of CML in chronic phase (CML-CP), by retroviral overexpression of BCR–ABL and by crossing BCR–ABL transgenic mice, revealed that Evi1 is predominantly enriched in the stem cell fraction and associated with an enhanced proliferative as well as a leukemia-initiating capacity and that Evi1-high CML-CP cells exhibit resistance to TKIs. Overexpressing BCR–ABL and NUP98–HOXA9 in Evi1-IRES-GFP knock-in mice to model CML in blast crisis (CML-BC), in which Evi1-high cells turned to be a major population as opposed to a minor population in CML-CP models, showed that Evi1-high CML-BC cells have a greater potential to recapitulate the disease and appear resistant to TKIs. Furthermore, given that Evi1 heterozygosity ameliorates CML-CP and CML-BC development and that the combination of Evi1 and BCR–ABL causes acute myeloid leukemia resembling CML-BC, Evi1 could regulate CML development as a potent driver. In addition, in human CML-CP cases, we show that *EVI1* is highly expressed in stem cell-enriched CD34+CD38–CD90+ fraction at single-cell level. This is the first report to clarify directly that Evi1-high leukemic cells themselves possess the superior potential to Evi1-low cells in oncogenic self-renewal, which highlights the role of Evi1 as a valuable and a functional marker of CML stem cells.

## Introduction

As multipotent hematopoietic stem cells (HSCs) reside at the apex of hematopoietic hierarchy, leukemic progeny from leukemic stem cells (LSCs) shape the bulk of the tumor with intact capacity of LSCs to self-renew. Physiological and biological similarities of LSCs to those of HSCs are supposed to be the main causes of the difficulties in establishment of LSC-targeted therapy.^1,2^

Chronic myeloid leukemia (CML) is a myeloproliferative disorder by *BCR*–*ABL*, which can transform HSCs into LSCs with a limitless capacity for self-renewal, whereas LSCs of acute myeloid leukemia (AML) are mainly composed of more differentiated progenitor cells.^3, 4, 5^ The relentless march of CML from chronic phase (CP) to blast crisis (BC) phase can result in fatal survival outcomes.^6^ Despite substantial prognostic improvement of CML by a specific debulking of tumor burden with a tyrosine kinase inhibitor (TKI) targeting ABL kinase, imatinib-treated CP patients can relapse and progress to BC because of the remnant CML stem cells.^7, 8, 9, 10^ Although recent findings have started to unveil the biological nature of CML stem cells,^11, 12, 13, 14, 15, 16^ further elucidation of the mechanisms controlling the self-renewal of these cells is still needed.

Defects in the molecular components that control hematopoiesis severely perturb normal development, one of which is ecotropic viral integration site 1 (Evi1), a predictor of poor outcomes in myeloid malignancies such as AML, myelodysplastic syndrome and CML-BC.^17, 18, 19, 20, 21^ In normal hematopoiesis, *Evi1* is restricted to embryonic and adult HSCs^22^ and cumulative data have placed *Evi1* as one of ‘stemness’ genes.^23, 24, 25^ The recent gene expression profiling of bulk samples or selective populations have shown that *EVI1* is one of LSC signature genes in AML and that stem cell-enriched CML CD34+ cells have high *EVI1*, underlining the relevance of EVI1 and LSCs.^26,27^ As EVI1 is an oncogenic transcription factor,^28, 29, 30, 31, 32, 33, 34^ prospective isolation of EVI1-high leukemic cells from clinical patients is unfeasible, so is the functional assessment of EVI1-high cells compared with EVI1-low cells intraindividually.

In this study, we aimed to cover in depth the regulation of CML stem cells by Evi1. Our single-cell analysis of primitive or differentiated subsets from primary CML-CP samples show that EVI1 is highly expressed in stem cell-enriched CD34+CD38–CD90+ cells. Furthermore, we have established multiple CML mice model with Evi1-Internal Ribosomal Entry Site (IRES)-green fluorescent protein (GFP) knock-in allele, in which Evi1-high CML cells can be separated directly and prospectively using a single GFP^35^ to evaluate their capacity for leukemia development. Evi1-IRES-GFP allele leukemia animals provide us for the first time with a more definite hierarchical map in CML hematopoiesis and, through loss- and gain-of-function studies we could evaluate the functional role of Evi1 in CML. This study could also determine whether Evi1-high CML cells could have resistance to TKI therapy.

## Results

### *EVI1* is highly expressed in human CML-CP stem cells

**Figure 1:**
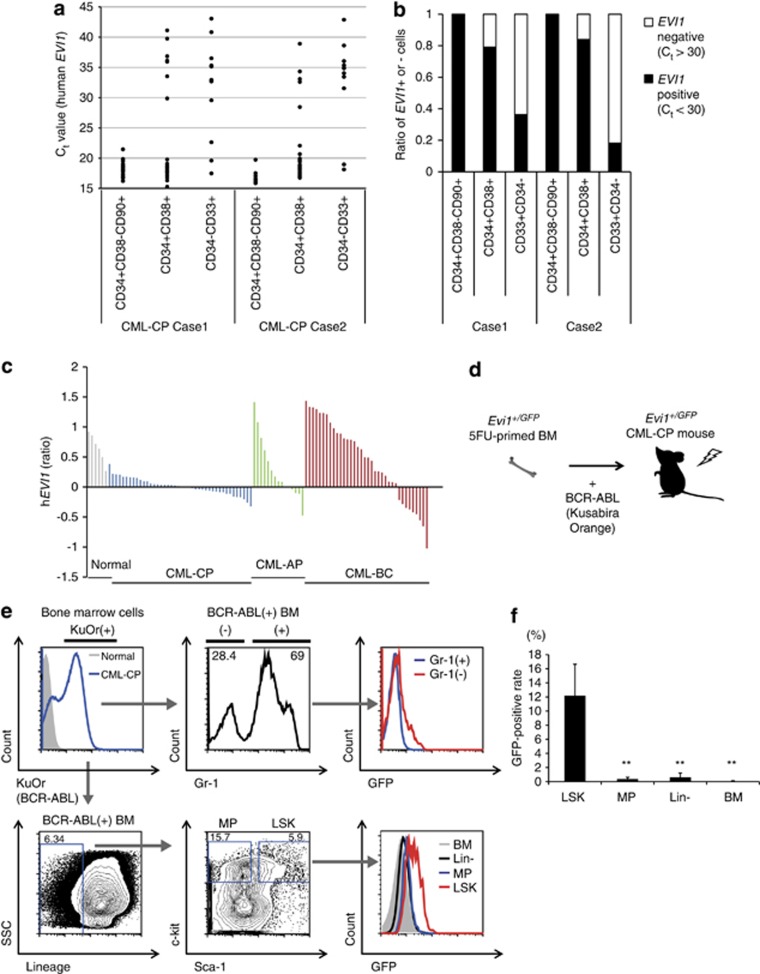
Both human and mouse CML-CP stem cells show high Evi1 expression. (**a**) BM cells from two CML-CP patients were single-cell sorted into CD34+CD38–CD90+ (*n*=21 for case 1, *n*=12 for case 2), CD34+CD38+ (*n*=24 for case 1, *n*=25 for case 2) and CD34–CD33+ fractions (*n*=11 for both cases) and *EVI1* was evaluated by quantitative real-time–PCR. *C*_t_ value was shown. (**b**) Ratio of *EVI1-*positive or -negative cells in each fraction from two CML cases. *C*_t_=30 was set as a threshold value of *EVI1* positivity. (**c**) Relative *EVI1* expression found to be upreguated in BM samples from advanced phase (AP, BC) disease compared with those from CP of CML. Microarray data from Radich *et al.*^1^ (*n*=6 for normal samples, *n*=42 for CML-CP, *n*=15 for CML-AP and *n*=36 for CML-BC). (**d**) Experimental design of Evi1-reporter CML-CP mice. 5FU-primed *Evi1*^*+/GFP*^ BM cells with retroviral BCR–ABL were transplanted into lethally irradiated recipient mice. (**e**) Representative FCM data of BM cells from Evi1-reporter CML-CP mice. In BCR–ABL (KuOr)-positive BM cells, Gr-1– CML cells had GFP-positive population, while almost Gr-1+ CML cells showed low GFP intensity (upper). When BCR–ABL (KuOr)-positive BM cells were analyzed with lineage markers, Sca-1 and c-kit, LSK population showed the highest GFP intensity (lower). (**f**) GFP-positive rates of stem/progenitor fractions and BM cells in Evi1-reporter CML-CP mice were shown (*n*=5). Data are mean±s.d. **P<0.001.

To ask whether CML-CP stem cells have high *EVI1*, we performed gene expression analysis in single primary CML-CP cells prospectively isolated from bone marrow (BM) samples of two newly diagnosed CML-CP patients (Supplementary Table S1). A total of 120 single cells (21 and 16 stem (CD34+CD38–CD90+) cells, 31 and 25 progenitor (CD34+CD38+) cells, 14 and 13 differentiated (CD34–CD33+) cells in cases 1 and 2, respectively) were subjected to single-cell gene expression analysis of housekeeping genes (*beta-actin* and *gapdh)*, *BCR*–*ABL* and *EVI1* (Supplementary Figure S1). As CML-CP cells could be distinguished by their positive expression of *BCR*–*ABL* from normal BM cells, it was revealed that, among CML-CP cells, CD34+CD38–CD90+ cells showed lower *C*_t_ values of *EVI1* than CD34+CD38+ cells or CD34–CD33+ cells intraindividually, implying the highest *EVI1* in CML-CP stem cells (Figure 1a). When *C*_t_=30 was set as a threshold of *EVI1* positivity, all CD34+CD38–CD90+ cells were *EVI1* positive (*C*_t_<30; *n*=21/21 for case 1, *n*=12/12 for case 2), while one-fifth of CD34+CD38+ cells (*n*=5/24 (20.8%), *n*=4/25 (16%), respectively) or three to four-fifths of CD34–CD33+ cells (*n*=7/11 (63.8%), *n*=9/11 (81.8%), respectively) were *EVI1* negative (*C*_t_>30) (Figure 1b). Microarray data from Radich *et al.*^1^ revealed that *EVI1* in the whole BM is upregulated in advanced phase (accelerated phase (AP) and BC) of CML compared with CML-CP, possibly implying the limited *EVI1* in CML-CP stem cells and the extended *EVI1* in BM of CML-AP and CML-BC (Figure 1c). These clinical data indicate a potential role of EVI1 as a valuable marker of CML-CP stem cells.

### Evi1-high CML-CP cells have LSK phenotype in murine CML model

To elucidate the *in vivo* expression pattern of Evi1 in CML-CP, 5-fluorouracil (5FU)-primed BM cells from heterozygous Evi1-IRES-GFP (*Evi1*^*+/GFP*^) knock-in mice were retrovirally transduced with BCR–ABL (detected with Kusabira Orange (KuOr) fluorescent protein) and injected into lethally irradiated recipient mice to generate ‘Evi1-reporter’ CML-CP mice (Figure 1d). Within BCR–ABL-positive (KuOr+) BM cells from moribund Evi1-reporter CML-CP mice (3-4 weeks after transplantation), the flow cytometry (FCM) analysis showed that immature (Gr-1-negative) CML cells have a higher GFP-positive rate than mature (Gr-1-positive) CML cells. GFP-positive rates of sub-populations in these mice revealed that Lineage-Sca-1+c-kit+ (LSK) fraction is the highest compared with myeloid progenitor (MP; Lineage-Sca-1-c-kit+) fraction, Lineage– (Lin–) fraction and whole BM cells (Figures 1e and f,Supplementary Figure S2a). Evi1-high KuOr+ cells comprised only 0.065% in the BM, were mostly Gr-1-, and about 90% of these cells showed LSK immunophenotype (Supplementary Figure S2b). The enlarged spleens from these mice also contained Evi1-high cells, which were the most abundant in LSK (Supplementary Figure S2c). Collectively, our data from human CML-CP samples and CML-CP mice revealed that Evi1 is highly expressed in the stem cell fraction.

### Evi1-high CML-CP LSK cells have a higher proliferative potential

**Figure 2:**
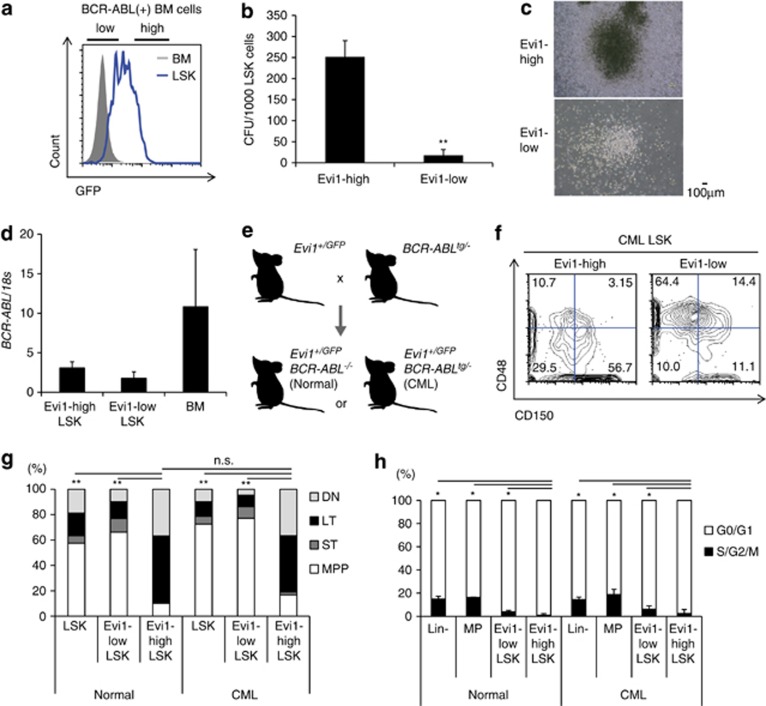
Evi1-high CML-CP LSK cells possess a higher clonogenic potential. (**a**) FCM panel of BM and LSK cells in Evi1-reporter CML-CP mice. The gate of Evi1 (GFP)-high fraction for separation was depicted. (**b**) Semisolid colony assay was done with 1000 Evi1-high or Evi1-low CML-CP LSK cells (*n*=7). (**c**) Images of colonies derived from Evi1-high (upper) or Evi1-low (lower) CML-CP LSK cells. (**d**) *BCR*–*ABL* mRNA expression was measured for each population from CML-CP mice (*n*=6). (**e**) Schematic abstract of Evi1-reporter CML transgenic mice. *Evi1*^*+/GFP*^ *BCR*–*ABL*^*tg/−*^ mice were used as CML model and *Evi1*^*+/GFP*^ *BCR*–*ABL*^*−/−*^ as normal controls. (**f**) Representative FCM data from Evi1-reporter CML transgenic mice. Within CML LSK cells, Evi1-high cells showed greater proportion of CD150+CD48- immunophenotype. (**g**) Immunophenotypic analyses of LSK cells with CD150 and CD48. Both Evi1-high normal and CML LSK cells mainly comprises CD150+CD48- (long-term HSC; LT) and CD150-CD48- (double negative; DN) fraction. CD150+CD48+ (short-term HSC; ST) and CD150-CD48+ (multipotent progenitor; MPP) fractions were commonly seen in Evi1-low LSK cells (*n*=7 mice per group). The differences between the proportions of LT in LSK cells were statistically evaluated. (**h**) Cell cycle status of each stem or progenitor fraction from normal or CML mice. Hoechst 33342 was measured by FCM (normal; *n*=3, CML; *n*=4). Data are mean±s.d. *P<0.01, **P<0.001, n.s., not significant.

As LSK cells showed a heterogeneous pattern of Evi1 in BM of Evi1-reporter CML-CP mice, we sorted the Evi1-high or Evi1-low fractions from KuOr+ LSK cells (Figure 2a). Evi1-high LSK cells showed a higher colony-forming potential *in vitro*, the sizes of which were larger than those from Evi1-low cells (Figures 2b and c). Co-culture of LSK cells with OP-9 cells revealed a higher clonogenic potential of Evi1-high LSK cells (Supplementary Figure S2e). Transcripts of *BCR*–*ABL* were comparable between Evi1-high and Evi1-low LSK populations (Figure 2d), suggesting little relevance of BCR–ABL to the enhanced proliferation of Evi1-high CML cells. These results suggest that Evi1-high CML-CP LSK cells have the proliferative advantage.

### Evi1-high CML LSK cells have SLAM LSK marker profile with CML-initiating potential and TKI resistance

Generating primary CML-CP mice by BCR–ABL retrovirus with irradiated recipients inevitably leads to damaged BM microenvironment and likely overestimation of extramedullary hematopoiesis. To clarify the precise behavior of Evi1-high CML LSK cells *in vivo*, we crossed *Evi1*^*+/GFP*^ mice with p210 BCR–ABL transgenic mice (*BCR*–*ABL*^*tg/−*^),^36^ which develop a CML-like disease (Supplementary Figure S3a), to analyze undamaged BM of CML-CP (Figure 2e). *Evi1*^*+/GFP*^ *BCR*–*ABL*^*tg/−*^ (CML) mice showed myeloid cell expansion in BM with mild splenomegaly (Supplementary Figures S3b and c) representing a myeloproliferative disorder phenotype of this model. The median survival of *Evi1*^*+/GFP*^ *BCR*–*ABL*^*tg/−*^ mice in our experiments was 287 days, consistent with the previous report,^36^ which revealed that Evi1-IRES-GFP allele had no unforeseen effect on CML development. In addition to the expanded bulk of CML BM cells, *Evi1*^*+/GFP*^ *BCR*–*ABL*^*tg/−*^ mice showed an increase in GFP intensity and the number of LSK cells (Supplementary Figures S2a and S3c). Evi1-high cells in the BM of these mice only amounted to 0.05% with negative marker profiles of Gr-1, B220, CD4, CD8, TER-119 and intermediate Mac-1, while almost all Evi1-low cells were Gr-1+Mac-1+. In Lin– cells, the majority of Evi1-high cells resided in Sca-1+c-kit+ fraction, consistent with the profile of a retroviral CML-CP model bearing *Evi1*^*+/GFP*^ allele (Supplementary Figure S3d). Strikingly, analysis of CD150/signaling lymphocyte activation molecule (SLAM) markers in LSK cells of *Evi1*^*+/GFP*^ *BCR*–*ABL*^*tg/−*^ mice clearly showed that Evi1-high LSK cells had a higher percentage of CD150+CD48– cells, a well-established marker phenotype of long-term stem cells (Figures 2f and g). Evi1-high CML LSK cells were the most in G0/G1 phase (quiescent) in CML setting, similar to the trend of sub-populations from normal *Evi1*^*+/GFP*^ mice (Figure 2h). Colony-forming assay showed the advantageous phenotype of Evi1-high CML LSK cells to proliferate *in vitro* (Supplementary Figure S3e). From these data, Evi1-high *Evi1*^*+/GFP*^ *BCR*–*ABL*^*tg/−*^ LSK cells had the most immature immunophenotype.

**Figure 3:**
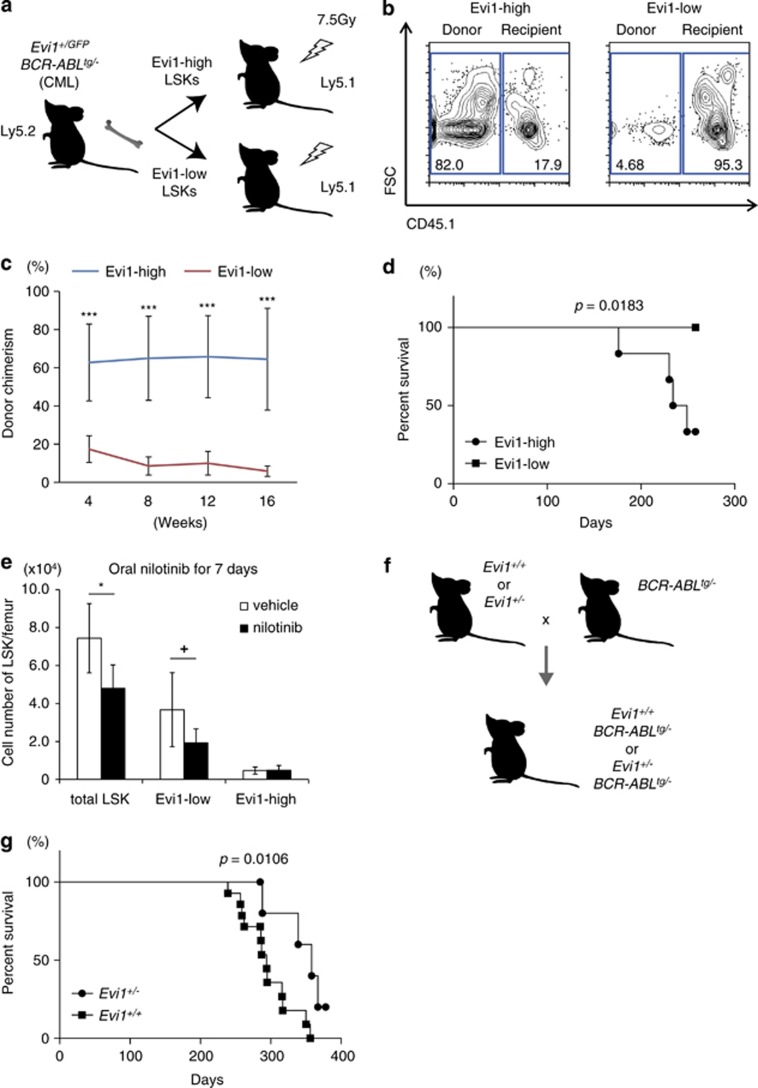
Evi1-high CML-CP cells show CML engraftment capacity and TKI resistance *in vivo*. (**a**) Design of non-competitive repopulation assay with Evi1-high or Evi1-low LSK cells from *Evi1*^*+/GFP*^ *BCR*–*ABL*^*tg/−*^ mice. (**b**) Representative FCM data of PB from recipients 16 weeks after BMT were shown to distinguish donor cells (CD45.2+CD45.1–) from recipient cells (CD45.2-CD45.1+). (**c**) Serial assessments of PB from recipients as for donor chimerism were done every 4 weeks after BMT (*n*=8 mice per group). (**d**) Kaplan–Meier plot of the survival of recipient mice transplanted with 5000 Evi1-high or Evi1-low LSK cells from *Evi1*^*+/GFP*^ *BCR*–*ABL*^*tg/−*^ mice (*n*=6 mice per group, *P*=0.0183). (**e**) Vehicle or nilotinib (75 mg/kg daily) were administered orally to *Evi1*^*+/GFP*^ *BCR*–*ABL*^*tg/−*^ mice for a week just after the CML development and the number of the residual LSK cells in femur were calculated (CML: with vehicle; CML nilo: with nilotinib; *n*=6 mice per group). (**f**) Schematic abstract of Evi1 knock-out CML transgenic mice. *Evi1*^*+/+*^ or *Evi1*^*+/−*^ mice were crossed with *BCR*–*ABL*^*tg/−*^ mice to generate *Evi1*^*+/+*^ *BCR*–*ABL*^*tg/−*^ or *Evi1*^*+/−*^ *BCR*–*ABL*^*tg/−*^ mice. (**g**) Kaplan–Meier plot of the survival of *Evi1*^*+/−*^ *BCR*–*ABL*^*tg/−*^ or *Evi1*^*+/+*^ *BCR*–*ABL*^*tg/−*^ mice (*n*=7 for *Evi1*^*+/−*^ *BCR*–*ABL*^*tg/−*^ mice and *n*=14 for *Evi1*^*+/+*^ *BCR*–*ABL*^*tg/−*^ mice, *P*=0.0106). Data are mean±s.d. ^+^*P*<0.05, *P<0.01, ***P<0.0001.

To evaluate the stem cell activity of Evi1-high CML LSK cells *in vivo*, we next performed BM transplantation (BMT) experiments, in which 5 × 10^3^ Evi1-high or Evi1-low LSK cells were injected intravenously into sublethally irradiated recipients (Figure 3a). FCM analyses of peripheral blood (PB) in recipients revealed the efficient engraftment of donor cells from Evi1-high LSK-transplanted cohort as contrasted with recipients of Evi1-low LSK cells (Figure 3b). The successive engraftment and multilineage reconstitution up to 16 weeks after BMT clearly indicated the higher repopulating capacity of Evi1-high LSK cells (Figure 3c, Supplementary Figure S3f). With such a prolonged engraftment, recipients with Evi1-high LSK cells died of CML (4 deaths per 6 mice), while those with Evi1-low LSK cells had no incidence of CML (0 death per 6 mice), revealing high CML-initiating potential of Evi1-high CML LSK cells (Figure 3d).

Drug resistance is one of critical characteristics of LSCs, which motivated us to conduct *in vivo* therapeutical interventions to *Evi1*^*+/GFP*^ *BCR*–*ABL*^*tg/−*^ mice. Oral administration of nilotinib, a potent BCR–ABL inhibitor, to *Evi1*^*+/GFP*^ *BCR*–*ABL*^*tg/−*^ mice for 7 days resulted in amelioration of BM cellularity and splenomegaly, which underlay the efficacy of the drug (Supplementary Figure S3c). In LSK fraction, although nilotinib-treated *Evi1*^*+/GFP*^ *BCR*–*ABL*^*tg/−*^ mice showed the reduced number of Evi1-low LSK cells in BM compared with vehicle-treated *Evi1*^*+/GFP*^ *BCR*–*ABL*^*tg/−*^ mice, that of Evi1-high LSK cells had no change irrespective of nilotinib treatment, which implied nilotinib resistance of Evi1-high CML cells (Figure 3e). The relatively nilotinib-resistant aspect of these cells was confirmed by *in vitro* colony-forming assay (Supplementary Figure S3g). FCM analysis of the residual cells in BM and spleen of nilotinib-treated *Evi1*^*+/GFP*^ *BCR*–*ABL*^*tg/−*^ mice revealed a marked increase in the percentage of CD150+CD48– fraction of Evi1-high LSK cells (Supplementary Figure S3h). The treatment had no impact on normal *Evi1*^*+/GFP*^ mice as to Evi1-positive rate (data not shown). These data of *Evi1*^*+/GFP*^ *BCR*–*ABL*^*tg/−*^ mice revealed CML stem cell activity and nilotinib resistance of Evi1-high cells.

### Evi1 heterozygosity impairs CML development

Based on these findings, we next crossed Evi1 heterozygous knock-out (*Evi1*^*+/−*^) mice with *BCR*–*ABL*^*tg/−*^ mice to clarify whether loss of Evi1 would affect CML development (Figure 3f). Surprisingly, 1-year follow-up revealed a significantly prolonged survival of *Evi1*^*+/−*^ *BCR*–*ABL*^*tg/−*^ mice (4 deaths per 7 cases), with an obvious contrast to the high lethality of *Evi1*^*+/+*^ *BCR*–*ABL*^*tg/−*^ mice (12 deaths per 14 cases; Figure 3g, *P*=0.0106). All dead mice in both cohorts had granulocytosis and mild splenoegaly (data not shown). Given that *Evi1*^*+/−*^ mice are fertile with no sign of BM failure over a year,^25^ these data suggest that Evi1 has a distinctive role in CML development.

### BCR–ABL and NUP98–HOXA9 induce myeloid BC of CML with Evi1 upregulation

**Figure 4:**
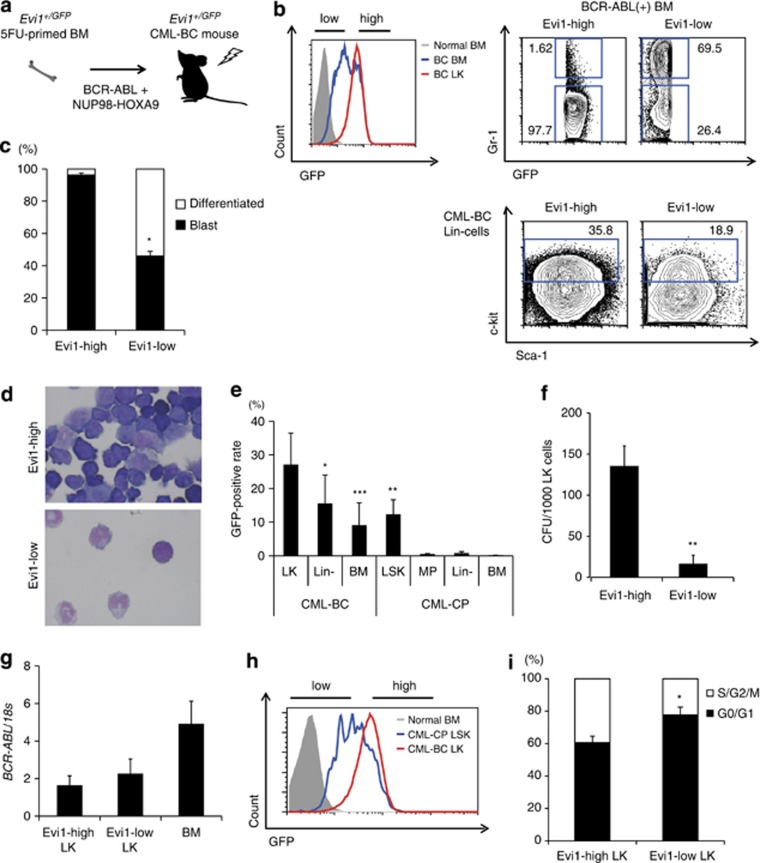
BCR–ABL with NUP98–HOXA9 augments Evi1 expression to cause CML-BC *in vivo*. (**a**) Experimental scheme of Evi1-reporter CML-BC mice. 5FU-primed *Evi1*^*+/GFP*^ BM cells with retroviral BCR–ABL and NUP98–HOXA9 were transplanted into lethally irradiated recipient mice. (**b**) GFP intensities of LK cells and BM cells from Evi1-reporter CML-BC mice were analyzed by FCM (upper left). The difference of Gr-1-positive rate in BCR–ABL (+) BM cells (upper right) and that of c-kit-positive rate in BCR–ABL (+) Lin– BM cells (lower) were shown. (**c**) The proportion of leukemic blasts in Evi1-high or Evi1-low CML-BC BM cells was shown (*n*=3). (**d**) Evi1-high or Evi1-low cells from CML-BC BM cells were sorted, cytospun and subjected to Wright–Giemsa staining. (**e**) GFP-positive rates of stem/progenitor fractions and BM cells in Evi1-reporter CML-BC or CP mouse were shown (*n*=13 for CML-BC mice, *n*=5 for CML-CP mice). (**f**) Semisolid colony assay was done with 1000 Evi1-high or Evi1-low CML-BC LK cells (*n*=5). (**g**) *BCR*–*ABL* mRNA expression was measured for each subset from CML-BC mice (*n*=3). (**h**) Representative FCM plot of LK cells from CML-BC mice (red) and LSK cells from CML-CP mice (blue). GFP intensity of normal BM cells is shown in solid gray. (**i**) Cell cycle status of Evi1-high or Evi1-low LK cells of CML-BC mice. Hoechst 33342 was measured by FCM (*n*=3). Data are mean±s.d. *P<0.01, **P<0.001, ***P<0.0001.

The BC phase of CML is characterized by high mortality and resistance to both TKI therapy and conventional chemotherapy, with HSC transplantation being the only therapy providing appreciable efficacy. Among CML-BC cases with high *EVI1*, there exist not only 3q-rearranged cases but also cases without 3q rearrangement, possibly suggesting unknown *EVI1* regulation. To evaluate a leukemogenic function of Evi1-high cells in CML-BC, BCR–ABL and NUP98–HOXA9 were co-transduced^37^ into 5FU-primed BM of *Evi1*^*+/GFP*^ mice to establish Evi1-reporter CML-BC mice (Figure 4a). Evi1-reporter CML-BC mice had an obviously high level of Evi1-positive cells in the BM compared with Evi1-reporter CML-CP mice (Figures 4b and e). Evi1-reporter CML-BC mice died at 14 days after BMT and showed emergence of blast cells in PB as well as in BM and spleen, a AML-like phenotype, which was markedly different from that of CML-CP model.

### Evi1-high CML-BC cells are enriched in the progenitor fraction

The morphological analyses of BM in Evi1-reporter CML-BC mice showed that Evi1-high cells mostly comprises leukemic blasts, while more than half of Evi1-low cells were differentiated cells (Figures 4c and d). From FCM analyses, most of Evi1-high CML-BC cells were negative for Gr-1, as opposed to Gr-1+ immunophenotype of Evi1-low cells. In Lin– cells of these mice, Evi1-high cells had a higher percentage of c-kit (Figure 4b). Totally, Evi1-high cells were rich in Lin-c-kit+ (LK) fraction of myeloid progenitor immunophenotype, the level of which was apparently higher than that of CML-CP (Figure 4e). Evi1-high LK cells had higher colony-forming capacity, indicating their proliferative advantage (Figure 4f, Supplementary Figure S4a). Transcripts of *BCR*–*ABL* and *HOXA9* were comparable between Evi1-high and Evi1-low LK populations (Figure 4g, Supplementary Figure S4b), reflecting little relevance of BCR–ABL or NUP98–HOXA9 dosage to the enhanced proliferation of Evi1-high cells. The majority of CML-BC LK cells showed higher Evi1 than CML-CP LSK cells, whereas CML-CP LSK cells had a wide range of Evi1, suggesting Evi1 upregulation in CML-BC model (Figure 4h). Cell cycle analysis revealed that Evi1-high LK cells were more in S/G2/M phase than their Evi1-low counterpart, possibly reflecting their higher proliferating potential (Figure 4i).

### Evi1-high CML-BC cells have higher leukemogenic potential and TKI resistance

**Figure 5:**
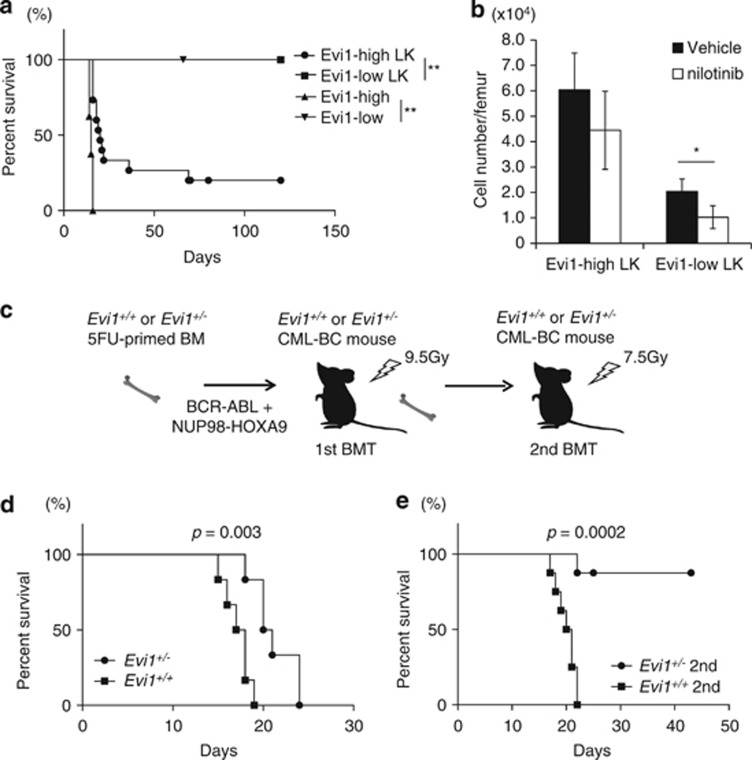
Evi1-high CML-BC cells possess leukemogenic potential and TKI resistance *in vivo*. (**a**) Kaplan–Meier plot of the survival of sublethally irradiated recipient mice receiving Evi1-high or Evi1-low CML-BC BM cells (*n*=8 mice per group) or mice with Evi1-high or Evi1-low LK cells of CML-BC (*n*=15 for Evi1-high CML-BC LK cells, *n*=9 for Evi1-low CML-BC LK cells). (**b**) Vehicle or nilotinib (75 mg/kg daily) were administered orally to Evi1-reporter CML-BC mice for a week from day 10 after BMT and the number of residual Evi1-high or Evi1-low CML-BC LK cells per femur were calculated (*n*=5). (**c**) Experimental scheme of generating *Evi1*^*+/+*^ or *Evi1*^*+/−*^ CML-BC mice. 5FU-primed *Evi1*^*+/+*^ or *Evi1*^*+/−*^ BM cells with retroviral BCR–ABL and NUP98–HOXA9 were transplanted into lethally irradiated recipient mice (1st BMT). BM cells from primary transplants were inoculated into sublethally irradiated recipient mice (2nd BMT). (**d**) Kaplan–Meier plot of the survival of primary transplants receiving 5FU-primed *Evi1*^*+/+*^ or *Evi1*^*+/−*^ BM cells transduced with BCR–ABL and NUP98–HOXA9 (*n*=6 mice per group). (**e**) Kaplan–Meier plot of the survival of secondary transplants with *Evi1*^*+/+*^ or *Evi1*^*+/−*^ CML-BC BM cells (*n*=8 mice per group). Data are mean±s.d. *P<0.01, **P<0.001.

To assess the leukemogenic potential of Evi1-high CML-BC cells *in vivo*, we performed serial BMT experiments. Recipients with 5 × 10^4^ Evi1-high CML-BC cells recapitulated the disease, while recipeints with Evi1-low cells showed no leukemia incidence. When 1 × 10^3^ LK cells were transplanted, leukemia-initiating cells were found to be enriched in the Evi1-high fraction with the frequency of 1/62 cells (1.6%), indicative of high leukemogenicity, whereas Evi1-low cells even in LK fraction had no potential for CML-BC development (Figure 5a, Supplementary Table S4). Under *in vitro* nilotinib treatment, Evi1-high LK cells showed a relatively higher proliferative capacity than Evi1-low cells (Supplementary Figure S4c). Seven-day *in vivo* nilotinib treatment resulted in the significant improvement of hepatosplenomegaly and only Evi1-low LK cells showed a significant decrease in number, indicating that Evi1-high LK cells are more resistant to TKI than the Evi1-low counterpart (Figure 5b,Supplementary Figure S4d). Even residual Evi1-high LK cells in BM after nilotinib still possessed leukemogenic potential (Supplementary Figure S4e). All of CML-BC mice treated with nilotinib relapsed with the expansion of Evi1-high cells after the drug cessation, reflecting a limited role of TKI for CML-BC (data not shown).

We next tested whether loss of Evi1 would affect the development of CML-BC. 5FU-primed *Evi1*^*+/+*^ or *Evi1*^*+/−*^ BM cells transduced with BCR–ABL and NUP98–HOXA9 were injected into lethally irradiated recipients (Figure 5c). All of the primary recipients developed CML-BC regardless of Evi1 dosage, with a slight delayed onset in *Evi1*^*+/−*^ cohort (Figure 5d). In the secondary recipients receiving 5 × 10^4^ CML-BC BM cells, however, *Evi1*^*+/−*^ CML-BC cells showed a significantly attenuated leukemogenic potential, whereas *Evi1*^*+/+*^ CML-BC retained the full capacity (Figure 5e). These results indicate that the development of CML-BC is dependent on Evi1 dosage.

### Combination of BCR–ABL and Evi1 induces AML

**Figure 6:**
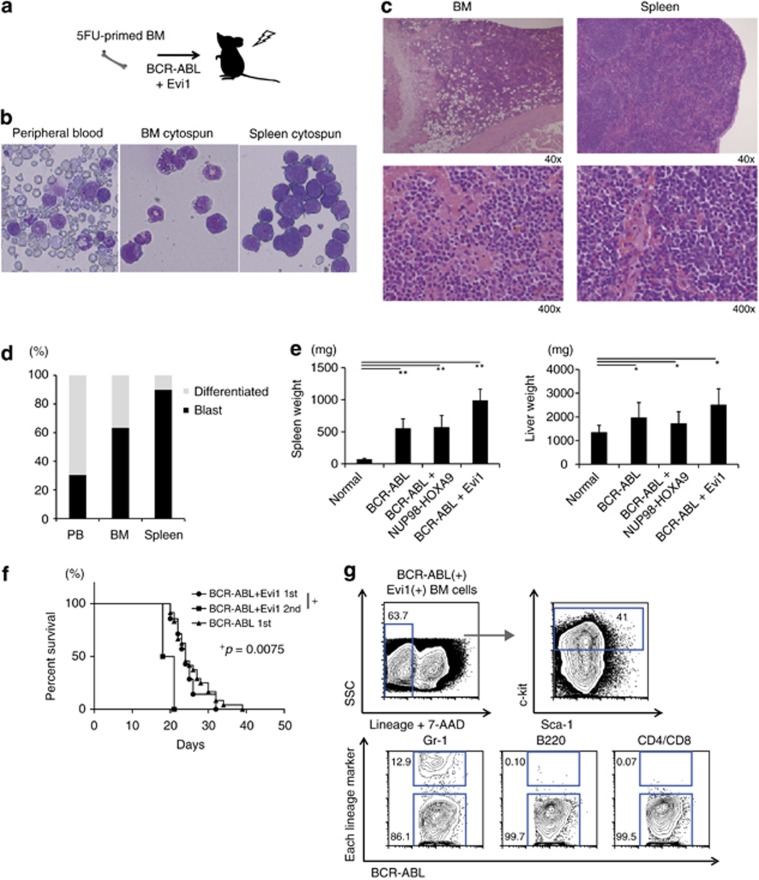
BCR–ABL cooperates with Evi1 to induce AML (CML-BC-like disease) *in vivo*. (**a**) Experimental scheme of BMT mice with BCR–ABL and Evi1 overexpression. 5FU-primed C57BL/6 BM cells with retroviral BCR–ABL and Evi1 were transplanted into lethally irradiated recipient mice. (**b**) Wright–Giemsa staining of PB, BM and spleen from BCR–ABL and Evi1 overexpressing mice. (**c**) HE staining of BM (upper) and spleen (lower) from BCR–ABL and Evi1 overexpressing mice with low (left) and high (right) magnitude. (**d**) The proportion of blasts in PB, BM and spleen from BCR–ABL and Evi1 overexpressing mice (*n*=3). (**e**) The weight of spleen and liver from normal (*n*=13), BCR–ABL (*n*=27), BCR–ABL+NUP98–HOXA9 (*n*=29) or BCR–ABL+Evi1 mice (*n*=3). (**f**) Kaplan–Meier plot of the survival of primary or secondary recipient mice transplanted with BCR–ABL and Evi1 overexpressing cells (*n*=7 for primary transplants and *n*=4 for secondary transplants) and primary recipient mice transplanted with BCR–ABL overexpressing cells (*n*=24). (**g**) Representative FCM data of BM cells from BCR–ABL and Evi1 overexpressing mice. In BCR–ABL(+) Evi1(+) BM cells, most cells were Lin– (upper left) and about 40% of Lin– cells had c-kit expression (upper right). Mostly BCR–ABL(+) Evi1(+) BM cells were Gr-1+, B220- and CD4/CD8− (lower). Data are mean±s.d. *P<0.01, **P<0.001.

Having established that Evi1 was upregulated *in vivo* by BCR–ABL and NUP98–HOXA9, we analyzed a role for Evi1 as a direct driver in CML-BC. Co-transduction of BCR–ABL and Evi1 caused lethality in transplanted mice (median survival: 24 days after BMT, Figures 6a and f). These recipients showed marked predominance of leukemic blasts in BM, spleen and PB, with a neutrophilic component, hypercellular BM and hepatosplenomegaly (Figures 6b–d). Splenomegaly was more obvious in the recipients of BCR–ABL plus Evi1 than those of either BCR–ABL alone or BCR–ABL plus NUP98–HOXA9, whereas liver sizes were comparable (Figure 6e). The disease was transplantable with the shorter latency and BM cells of the recipients with BCR–ABL and Evi1 were skewed to the myeloid lineage (Gr-1+) and Lin-c-kit+ cells were dominant in the primitive fractions (Figures 6f and g). Based on the criteria of Bethesda proposals,^38^ the disease was finally diagnosed as ‘Myeloid leukemia with maturation’. Thus, even without NUP98–HOXA9, activation of Evi1 can induce AML, resembling myeloid BC in CML, in collaboration with BCR–ABL in mice. Given that the duration of CML-CP development by BCR–ABL retrovirus is about 3–4 weeks, and Evi1 alone could induce myelodysplastic syndrome/AML in mice after 6 months or more,^39,40^ these results indicate that Evi1 can have a causative role in blastic transformation of CML.

## Discussion

Discovery strategies aimed to identify novel driver oncogenic lesions have succeeded in enrichment of the catalog of therapeutic targets, the most striking of which is *BCR*–*ABL* where TKI therapy has conferred tremendous benefits to CML patients with a sustained debulking of tumor burden. Even in the era of TKI treatment in CML, blastic transformation can occur with the translocation involving *EVI1* locus,^41,42^ which is crucial because the clinical outcomes of CML plummet from CP (80%) to BC (20%).^43,44^ Allogeneic HSC transplantation is so far the only potential remedy for high *EVI1* cases of CML-BC as well as AML, emphasizing the need for new therapy targeting EVI1.^18,21,45,46^

Our hypothesis stems from the fact that EVI1 is a valuable prognostic factor of myeloid malignancies as well as a critical regulator of HSCs. To overcome the incapability of prospective separation of intraindividual EVI1-high cells in human leukemias, we used our original *Evi1*^*+/GFP*^ mice, developing two types of CML-CP model to show that Evi1 is a valuable marker for CML stem cells. Both in retrovirally developed CML-CP mice and *BCR*–*ABL*^*tg/−*^ mice, Evi1 is restricted to a small population with primitive immunophenotypic markers, especially in the latter, the most primitive profile like SLAM-LSK represents Evi1-high cells. When seen against previous studies,^47, 48, 49^ the novelty of this study lies in that high Evi1 could distinguish CML-CP stem cells even from SLAM-LSK cells or non-SLAM-LSK cells. This point can be translated to human CML cases like that high *EVI1* means the increasing number of CML stem cells. Evi1-high CML LSK cells have a superior proliferative potential *in vitro*, a superior leukemia-initiating capacity *in vivo* and nilotinib resistance. The resistant aspect of Evi1-high cells to nilotinib would fit into clinical data that high *EVI1* is related to TKI resistance.^17^ The *in vivo* quiescent status of Evi1-high CML-CP cells, which could proliferate aggressively *in vitro*, may be controlled by hypoxic BM niche microenvironment.^50,51^ In line with less dependence of CML stem cells on BCR–ABL,^2,52,53^ Evi1-high CML-CP LSK cells showed a comparable *BCR*–*ABL* to their Evi1-low counterpart, reflecting little addiction to BCR–ABL (Figure 2d).

In accordance with Evi1-reporter CML-CP model, our single-cell analysis of clinical CML-CP cases revealed the highest *EVI1* in stem cell-enriched CD34+CD38–CD90+ cells, reflecting CML-CP as a stem cell disease (Figure 1a). Using *BCR*–*ABL*-specific primers, we could effectively distinguish CML-CP cells (*BCR*–*ABL*+) from remnant normal cells (*BCR*–*ABL*–). These human data could reinforce the value of murine CML data.

We also examined retroviral Evi1-reporter CML-BC mice and showed that a sizable fraction of LK cells have distinct Evi1 expression in sharp contrast to the CML-CP model. CML-BC stem cells are exclusively enriched in Evi1-high LK cells with resistance to nilotinib. The impact of combining the CML-BC with *Evi1* upregulation is in strong contrast to that of the simple BCR–ABL BMT model, which only leads to CML-CP in mice 3–4 weeks after inoculation (Figure 4h). Taken together, this is the first report that visualizes *Evi1* upregulation in *in vivo* leukemia models. The finding is consistent with the previous report that showed the transcriptional regulation of *Evi1* by NUP98–HOXA9 *in vitro*.^54^ In our CML-BC model, *HOXA9* showed no difference between Evi1-high and Evi1-low LK cells, eliminating the possible dependence of Evi1-high cells on NUP98–HOXA9 (Supplementary Figure S4b).

In our Evi1 knock-out studies of CML-CP and BC mice, Evi1 heterozygosity alleviated the disease (Figures 3g and 5e), highlighting a functional role of Evi1 in CML pathogenesis. Although complete loss of Evi1 causes embryonic lethality in mice,^25,55^ *Evi1*^*+/−*^ mice have no sign of BM failure over a year and are fertile with a decreased size and function of HSCs.^25,35^ EVI1 has been reported to be relevant to BCR–ABL tyrosine kinase activity.^27^ Collectively, it is supposed that Evi1 reduction may permit the reversal of CML at the partial expense of HSCs.

We extended this study to establish a new model of AML by BCR–ABL and Evi1. It is meaningful that overexpression of Evi1 itself is the driver in blastic transformation of CML not only by Evi1-related fusions. Cuenco *et al.*^56^ have previously reported that EVI1 with BCR–ABL induces not leukemias but a fatal myeloproliferative disorder in mice. In contrast to the usage of human *EVI1* complementary DNA (in MSCV retroviral vector) in murine BMT model of Cuenco’s study, we utilized murine *Evi1* for Evi1 overexpression. Murine *Evi1* especially in pMYs vector is suitable for establishing myeloid leukemia in mouse BMT model (Jones *et al.*^42^ and unpublished), which resulted in a new AML model by Evi1 plus BCR–ABL. These findings can remind us of the importance of controlling Evi1 to impair CML-BC development by BCR–ABL and NUP98–HOXA9. As authentic Evi1 targets,^22,33,34^ such as Gata2, Pten and Pbx1, showed no difference in expression levels between Evi1-high and Evi1-low CML cells (data not shown), the further exploration of CML-specific Evi1 targets would be warranted.

As opposed to Evi1-reporter CML models, in AML model by MLL-ENL retrovirus, Evi1-high MLL-ENL leukemic cells showed no advantage in leukemia initiation compared with Evi1-low cells (Supplementary Figure S5). Other Evi1-reporter AML models by MOZ-TIF2 and TP+AE never generated Evi1-high fraction, suggesting the high affinity of Evi1 for stem cell disease such as CML. Although Evi1 could not enrich MLL-ENL AML LSCs in our model, it is possible that Evi1 reduction in the bulk leukemia cells would be a key in amelioration of MLL-related leukemia as previously investigated.^25^ Exact introduction of these oncogenes to HSCs by different approaches such as via a transgene or a knocking-in technology could unravel the relation between AML stem cells and Evi1.

In conclusion, high Evi1 can define the population of CML stem cells that are resistant to nilotinib. This is the first report that uncovers the importance of leukemia cells with high Evi1 intraindividually. Combinatorial analyses of Evi1-IRES-GFP allele CML animals and single cells from primary CML-CP patients covered in depth the critical regulation of CML stem cells by Evi1. To elucidate the complex cellular circuits involving Evi1, a further genetic and epigenetic investigation of Evi1-high cells would unveil precise mechanisms of the phenotype in leukemias.

## Materials and methods

### Single-cell gene expression analysis

Primary BM samples of CML-CP patients were stained with anti-CD34, anti-CD38, anti-CD33 and anti-CD90 (Supplementary Table S2), and single-cell sorted by FACSAriaII (BD Biosciences, San Jose, CA, USA) gating on each population excluding cell duplets directly into individual wells of 96-well plates filled with 5 μl RT/Specific Target Amplification master mix solution (Fluidigm, San Francisco, CA, USA) immediately followed by reverse transcription and gene-specific pre-amplification with CellsDirect One-Step qRT-PCR kit (Life Technologies, Carlsbad, CA, USA) in the same plates. Each complementary DNA was subjected to quantitative real-time PCR by LightCycler 480 (Roche, Basel, Switzerland) using SYBR Green (TAKARA, Otsu, Japan) with the primers inside of the pre-amplified complementary DNA. Gene-specific primers were listed in Supplementary Table S3. Only samples with a specific product peak in the melting curve analysis of housekeeping genes were taken for further analysis. This study was approved by ethical committee of the University of Tokyo.

### Population quantitative real-time–PCR

Target gene expression for bulk samples or sorted populations was evaluated by LightCycler 480 as described previously.^35^ All assays were performed in triplicate and expression levels relative to *18s ribosomal RNA* were determined. Primer sequences are listed in Supplementary Table S3.

### Mice

Evi1-IRES-GFP knock-in (*Evi1*^*+/GFP*^) mice, Evi1 knock-out (*Evi1*^*+/−*^) mice and p210 BCR–ABL transgenic (*BCR*–*ABL*^*tg/−*^) mice on a C57BL/6 (Ly5.2) background were used and genotyped as previously described.^25,35,57^ CML development of *BCR*–*ABL*^*tg/−*^ mice was confirmed by leukocyte elevation (>10 000 cells/μl) and >80% increase of Gr-1+ cells in PB. Ly5.1 mice were purchased from SRL Inc. (Tokyo, Japan). All mice were kept at the Animal Center for Biomedical Research of the University of Tokyo.

### Flow cytometry

FACSAriaII was used for cell sorting of stained cells and LSRII (BD Biosciences) was used for other analyses. Data were analyzed with FlowJo (TreeStar, Ashland, OR, USA). In experiments with the Evi1-IRES-GFP knock-in mouse, a ‘fluorescence minus one’ littermate control was analyzed in parallel to set GFP gates.^35^ Antibodies are listed in Supplementary Table S2.

### Retrovirally developed leukemia model mice

Plat-E packaging cells^58^ were transiently transfected with retroviral constructs by FuGENE6 (Roche). 5FU-primed BM cells were incubated in RPMI1640 medium (Wako, Osaka, Japan) with cytokines (50 ng/ml stem cell factor, 50 ng/ml thrombopoietin, 10 ng/ml interleukin-6) for 24 h as previously described,^25,35^ and cultured cells were infected with retrovirus on RetroNectin (TAKARA)-coated plate. The combination of donor cells and retroviruses is as follows; *Evi1*^*+/GFP*^ BM with pGCDNsam/BCR–ABL/IRES-KuOr for *Evi1*^*+/GFP*^ CML-CP, *Evi1*^*+/GFP*^ BM with pGCDNsam/BCR–ABL/IRES-KuOr and pMSCVpuro/NUP98–HOXA9 for *Evi1*^*+/GFP*^ CML-BC, *Evi1*^*+/GFP*^ BM with pGCDNsam/MLL-ENL/IRES-KuOr or pGCDNsam/MOZ-TIF2/IRES-KuOr or pMSCV/TEL-PDGFR/IRES/AML1-ETO for *Evi1*^*+/GFP*^ AML, and C57BL/6 (Ly5.2) BM with pGCDNsam/BCR–ABL/IRES-GFP and pGCDNsam/Evi1/IRES-KuOr for a new AML model. The infected cells were harvested 48 h after retrovirus infection, and injected into lethally (9.5 Gy) irradiated recipient mice in competition with 2 × 10^5^ unfractionated BM cells from congenic mice. BCR–ABL-positive CML cells were distinguished by each fluorescent protein.

### *In vivo* transplantation assay

For secondary BMT assay of BCR–ABL and NUP98–HOXA9 overexpressing mice and BCR–ABL and Evi1 overexpressing mice, sublethally irradiated (7.5 Gy) mice (Ly5.2) were injected with the indicated subsets from these mice (Ly5.2). Reconstitution of donor-derived cells was monitored by GFP or KuOr. For BMT assay of *BCR*–*ABL*^*tg/−*^ mice, sublethally irradiated mice (Ly5.1) were injected with the indicated subsets from *Evi1*^*+/GFP*^ *BCR*–*ABL*^*tg/−*^ mice (Ly5.2). Reconstitution was monitored by Ly5.1, CD4, CD8, B220, TER-119, Mac-1 and Gr-1.

### Nilotinib treatment

For *in vivo* treatment, nilotinib (AMN107-AA) was diluted at 10 mg/ml in 10% 1-methyl-2-pyrrolidone (Sigma-Aldrich, St Louis, MO, USA), 90% polyethylenglycol 300 (Sigma-Aldrich) and administered by oral gavage at 75 mg/kg once a day for a week. A mixture without nilotinib was used as vehicle. For *in vitro* treatment, nilotinib diluted in dimethylsulphoxide (Sigma-Aldrich) was used at the final concentration of 1 or 5 μmol/l.

### Colony-forming assay

Cells (1 × 10^3^) were plated into MethoCult GF M3434 (StemCell Technologies, Vancouver, BC, Canada) as described previously.^59^ The number of colonies was counted at day 7. Images were taken with a Nikon Eclipse TE2000-U (Nikon, Tokyo, Japan).

### Hoechst 33342 staining

Cells were incubated with 5 ng/ml Hoechst 33342 (Invitrogen/Life Technologies) and 25 μg/ml verapamil at 37 °C for 45 min.^35^

### OP-9 co-culture

OP-9 cells were pre-seeded on 24-well plates at day –1.^59,60^ One hundred LSK cells from Evi1-reporter CML-CP mice were cultured from day 0 in alpha-minimum essential medium with 20% fetal calf serum, 1% penicillin+streptomycin, 2 μmol/l L-glutamine (Gibco/Invitrogen/Life Technologies), 1 μmol/l sodium pyruvate and 50 μmol/l 2-mercaptoethanol (Sigma-Aldrich). Culture medium was replaced every 4 days and images were taken at day 10.

### Statistical analysis

Statistical significance of differences between parameters was assessed by a two-tailed unpaired *t*-test. The overall survival of mice was analyzed with a Mantel–Cox test according to the Kaplan–Meier method.
