# Supplementary figures and images for: Evi1 defines leukemia-initiating capacity and tyrosine kinase inhibitor resistance in chronic myeloid leukemia
Source: Oncogene. 2014 Apr 21;33(42):5028–38. doi: 10.1038/onc.2014.108 (PMC4217142; doi:10.1038/onc.2014.108)

Supplementary Figure S1. Sato et al.

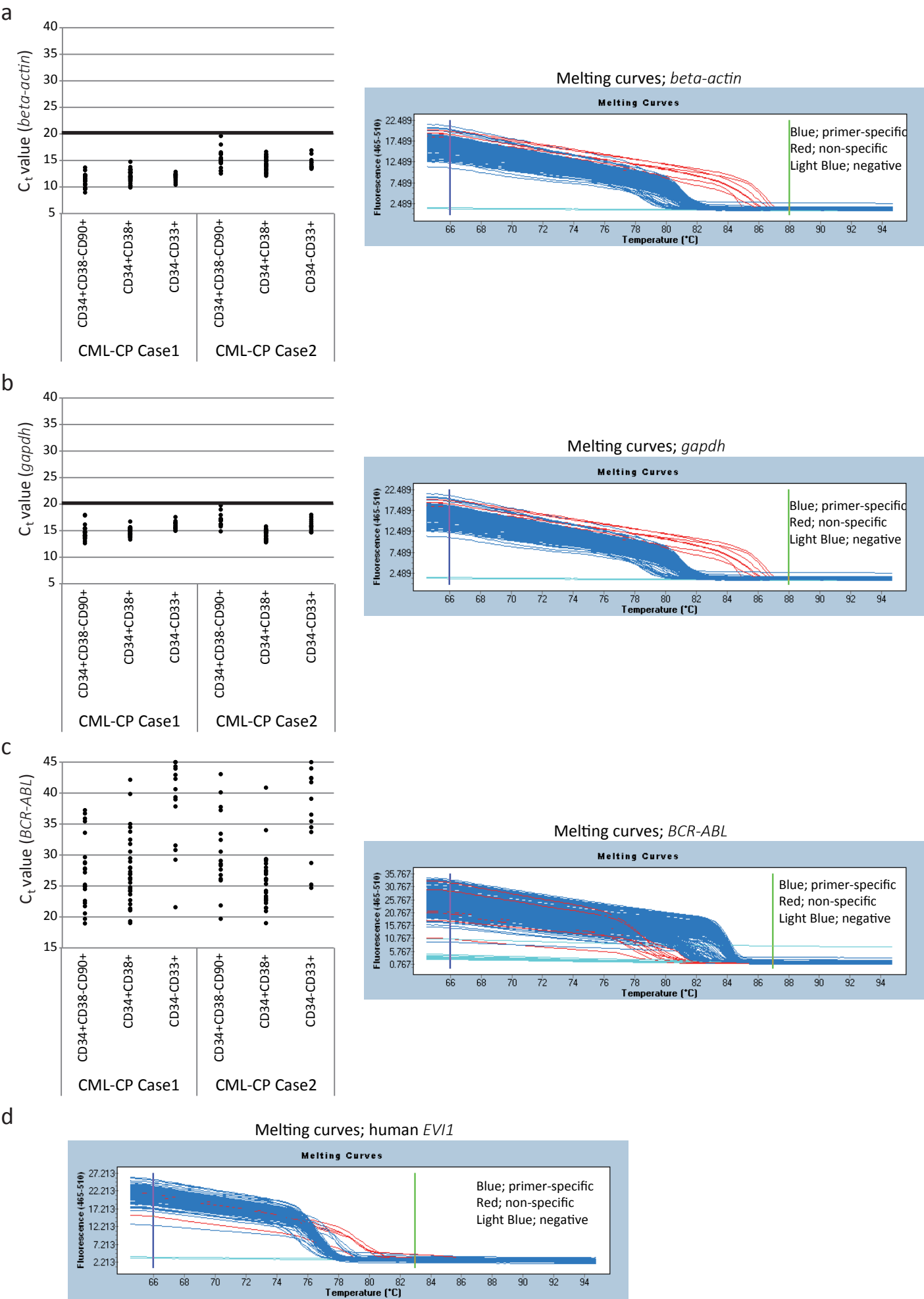

Supplement: Supplementary Figure S1 [file onc2014108x1.pdf]

Supplementary Figure S2. Sato et al.

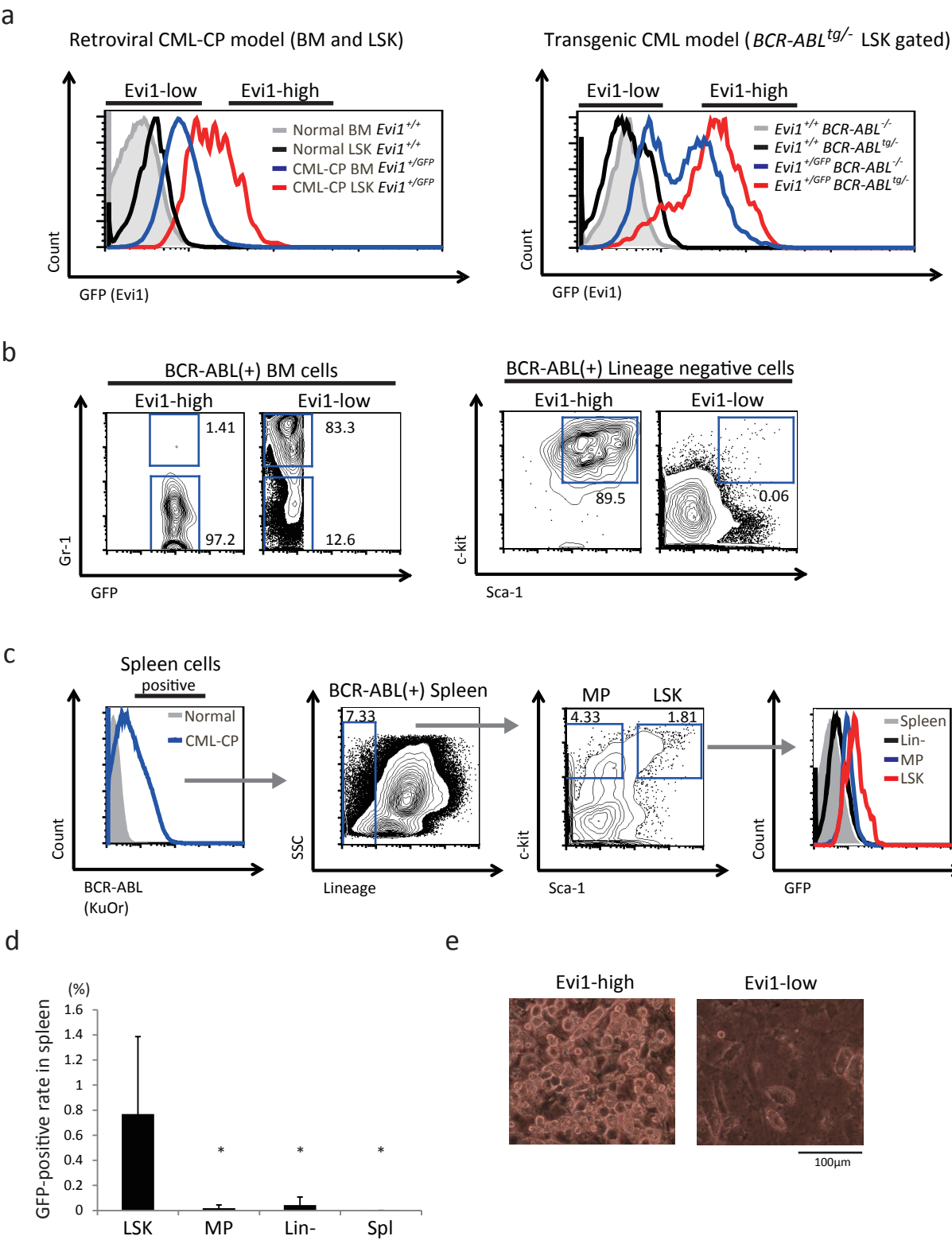

Supplement: Supplementary Figure S2 [file onc2014108x2.pdf]

Supplementary Figure S3. Sato et al.

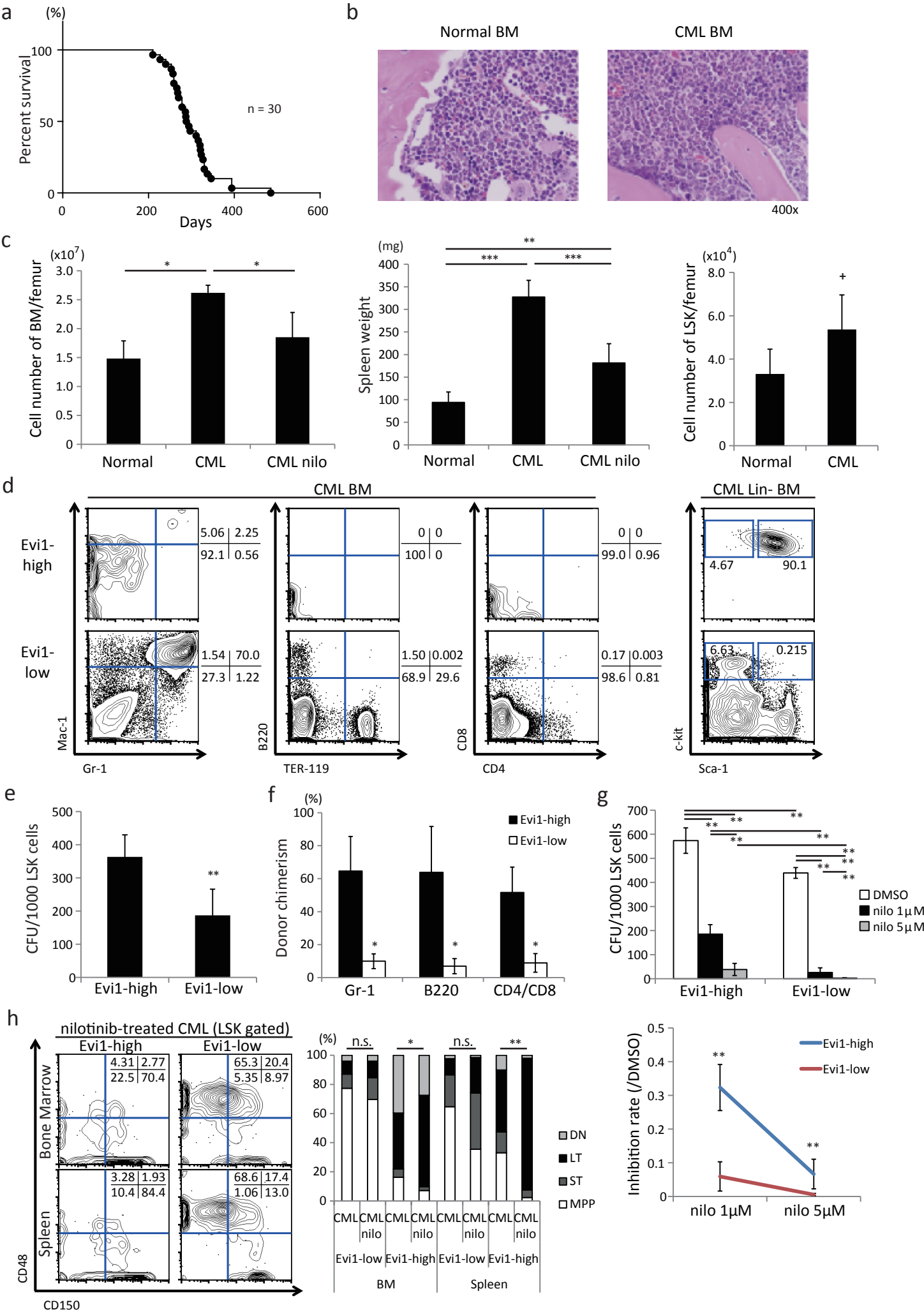

Supplement: Supplementary Figure S3 [file onc2014108x3.pdf]

Supplementary Figure S4. Sato et al.

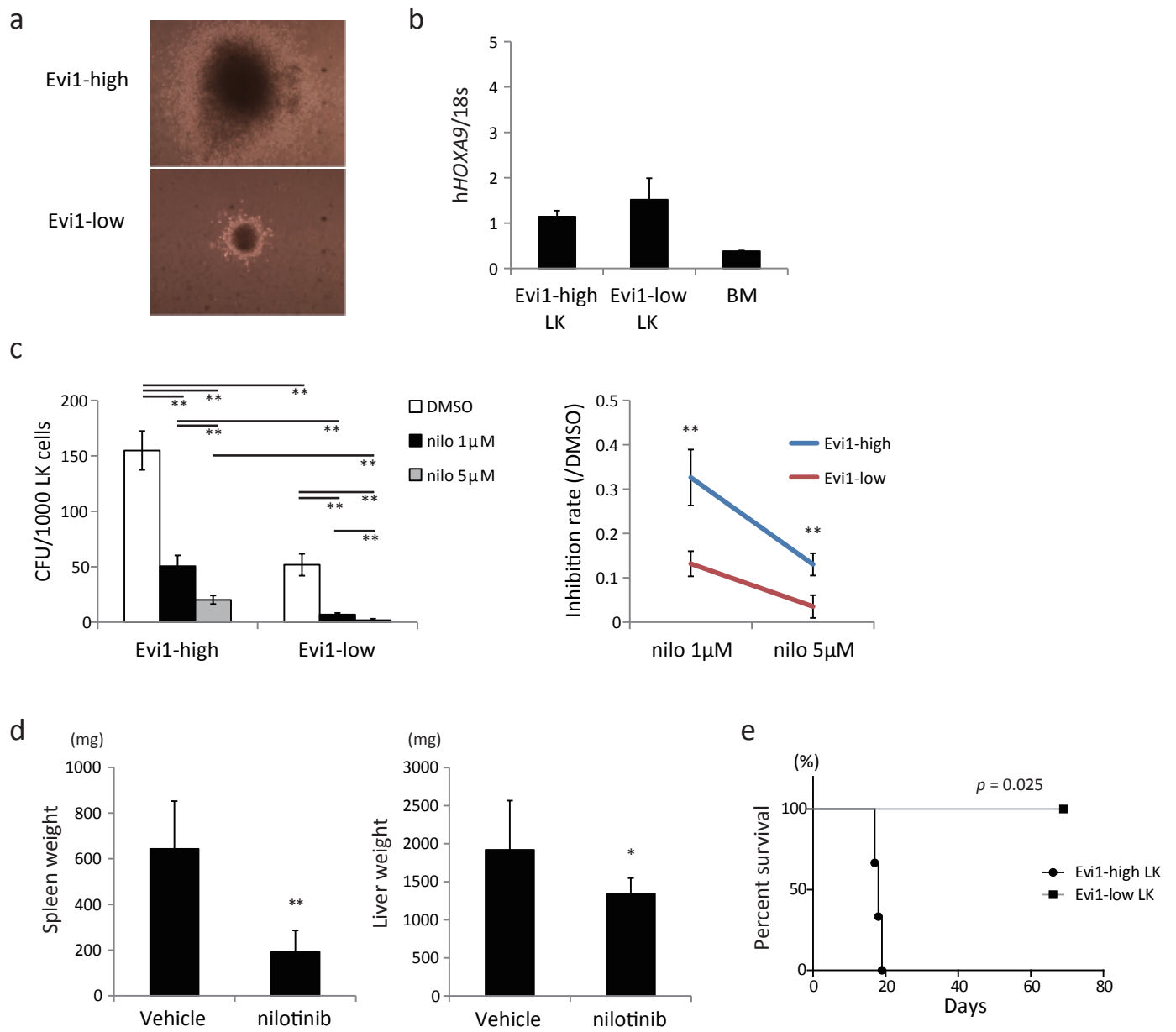

Supplement: Supplementary Figure S4 [file onc2014108x4.pdf]

Supplementary Figure S5. Sato et al.

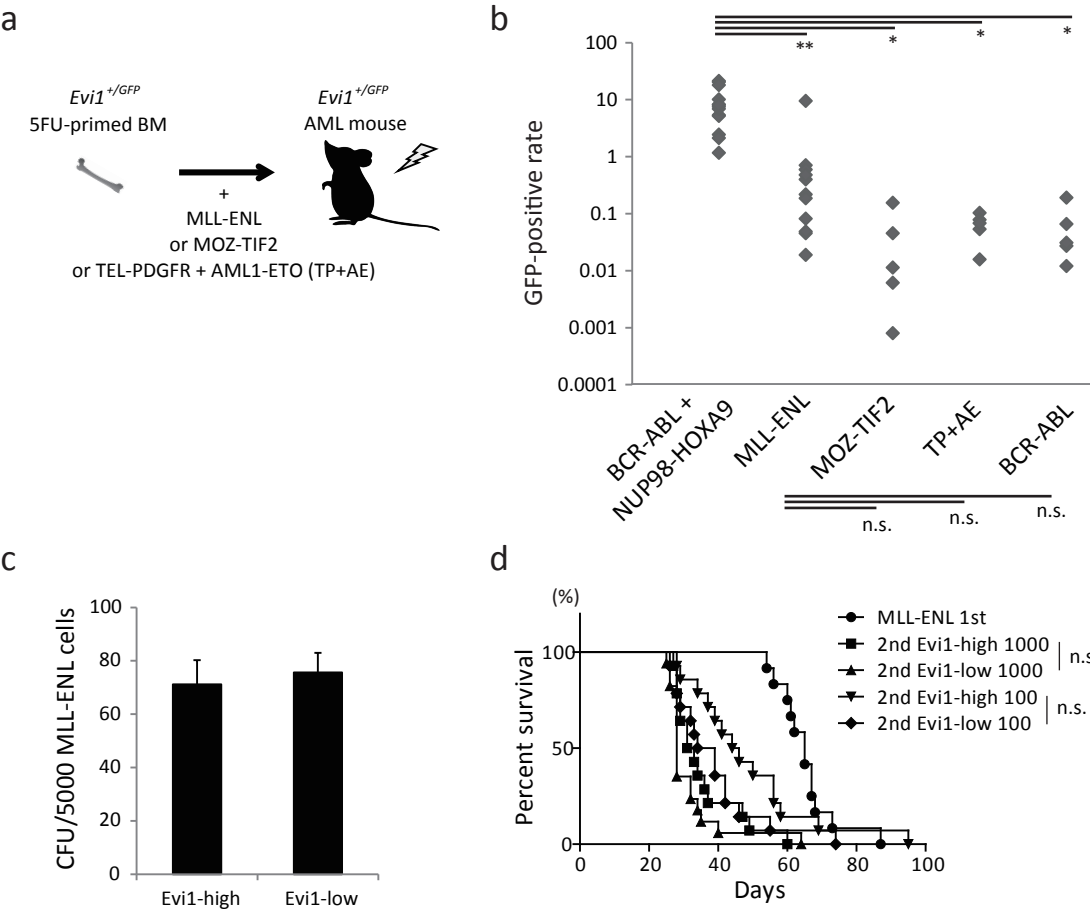

Supplement: Supplementary Figure S5 [file onc2014108x5.pdf]
