# Supplementary material for: Evi1 defines leukemia-initiating capacity and tyrosine kinase inhibitor resistance in chronic myeloid leukemia
Source: Oncogene. 2014 Apr 21;33(42):5028–38. doi: 10.1038/onc.2014.108 (PMC4217142; doi:10.1038/onc.2014.108)
Supplement: Supplementary Information [file onc2014108x6.doc]

**Supplementary Information**

**Evi1 Defines Leukemia-initiating Capacity and Tyrosine Kinase Inhibitor Resistance in Chronic Myeloid Leukemia**

Tomohiko Sato, Susumu Goyama, Keisuke Kataoka, Ryo Nasu, Takako Tsuruta-Kishino, Yuki Kagoya, Arika Nukina, Katsuyoshi Kumagai, Naoto Kubota, Masahiro Nakagawa, Shunya Arai, Akihide Yoshimi, Hiroaki Honda, Takashi Kadowaki and Mineo Kurokawa

This file includes four supplementary tables and five supplementary figures with figure legends.

**Supplementary Tables**

**Supplementary Table S1**

**Patient information**

Clinical information of two CML-CP patients (CML-CP Case 1, 2) is listed. Both of CML-CP patients had Philadelphia 1 (Ph1) chromosome with 100% in G-banding.

| **Case No.** | **Age** | **Gender** | **Diagnosis** | **Ph1 (G-band)** | **FISH BCR-ABL+** | **BCR-ABL type** |
| --- | --- | --- | --- | --- | --- | --- |
| CML-CP 1 | 75 | F | CML-CP | 20/20 (100%) | 192/200 (96%) | p210(b2a2) |
| CML-CP 2 | 66 | M | CML-CP | 20/20 (100%) | 188/200 (94%) | p210(b2a2) |

**Supplementary Table S2**

**A list of antibodies for flow cytometry**

Each table shows antibodies used for mouse cells or human cells.

1. Mouse

| **Epitope** | **Clone** | **Fluorophore** | **Supplier** |
| --- | --- | --- | --- |
| Gr-1 | RB6-8C5 | Biotin | BioLegend |
|  | RB6-8C5 | PE | eBioscience |
|  | RB6-8C5 | APC | BioLegend |
| Mac-1 | M1/70 | Biotin | BioLegend |
|  | M1/70 | APC | BioLegend |
| B220 | RA3-6B2 | Biotin | BioLegend |
|  | RA3-6B2 | APC | BioLegend |
| TER-119 | TER-119 | Biotin | BioLegend |
|  | TER-119 | PE | BioLegend |
| CD3ε | 145-2C11 | Biotin | BioLegend |
| CD4 | GK1.5 | Biotin | BioLegend |
|  | RM4-5 | PE | eBioscience |
|  | GK1.5 | APC | BioLegend |
| CD8a | 53-6.7 | Biotin | BioLegend |
|  | 53-6.7 | APC | BioLegend |
| CD127 | A7R34 | Biotin | BioLegend |
| Sca-1 | E13-161.7 | PE-Cy7 | BioLegend |
| c-kit | 2B8 | APC | BioLegend |
|  | 2B8 | PE-Cy5 | BioLegend |
| CD48 | HM48-1 | PE | BioLegend |
| CD150 | TC15-12F12.2 | APC | BioLegend |
| CD45.1 | A20 | PE | BD Pharmingen |

1. Human

| **Epitope** | **Clone** | **Fluorophore** | **Supplier** |
| --- | --- | --- | --- |
| CD34 | 581 | PE | BioLegend |
| CD38 | HIT2 | FITC | BioLegend |
| CD90 | 5E10 | Alexa 647 | BioLegend |
| CD33 | P67.6 | PE-Cy7 | BD Pharmingen |

**Supplementary Table S3**

1. A list of primers for single-cell quantitative RT-PCR (qRT-PCR)

| **Gene** | **Application** | **Forward** | **Reverse** |
| --- | --- | --- | --- |
| *EVI1* | Pre-amp | GACCAAGTTTTTCCTGATTTGC | CCCTCTCTTCAGTATGTGACAGC |
| *EVI1* | qRT-PCR | CCAAGTTTTTCCTGATTTGCAAAGC | CCTCTCTTCAGTATGTGACAGCA |
| *BCR-ABL* | Pre-amp | CAGAGAGAGAAGAGGGCGAA | GACCCAGCCTTGGCCATTTT |
| *BCR-ABL* | qRT-PCR | AACTCCAGACTGTCCACAGCA | AACGAGCGGCTTCACTCA |
| *beta-actin* | Pre-amp  /qRT-PCR | CCAACCGCGAGAAGATGAC | TAGCACAGCCTGGATAGCAA |
| *Gapdh* | Pre-amp  /qRT-PCR | ACACCATGGGGAAGGTGAAG | GTGACCAGGCGCCCAATA |

Primers used for pre-amplification (Pre-amp) of each target gene and primers for single-cell qRT-PCR are listed.

1. A list of primers for population qRT-PCR

Primers used for qRT-PCR of each BM population from leukemic mice are listed.

| **Gene** | **Species** | **Forward** | **Reverse** |
| --- | --- | --- | --- |
| *BCR-ABL* | human | CCACTGGATTTAAGCAGAGTTCA | AACGAGCGGCTTCACTCAGA |
| *HOXA9* | human | CCACGCTTGACACTCACACT | CAGTTCCAGGGTCTGGTGTT |
| *18s RNA* | mouse | GTAACCCGTTGAACCCCATT | CCATCCAATCGGTAGTAGCG |

**Supplementary Table S4**

**Limiting dilution assays of CML-BC cells**

To evaluate leukemia-initiating capacity of Evi1-high CML-BC cells, sublethally irradiated recipient mice were transplanted with 5, 50, 500, 5000 or 50000 Evi1-high CML-BC cells (n = 4 mice per group). The rate of death by CML-BC was listed for each recipient group.

| **Cells** | **Cell number** | **Death** | **Cause** |
| --- | --- | --- | --- |
| Evi1-high | 50000 | 4/4 | CML-BC |
|  | 5000 | 4/4 | CML-BC |
|  | 500 | 4/4 | CML-BC |
|  | 50 | 3/4 | CML-BC |
|  | 5 | 0/4 | - |
| Evi1-low | 50000 | 0/4 | - |

**Supplementary Figure legends**

**Supplementary Figure S1.**

BM cells from two CML-CP patients were single-cell sorted into CD34+CD38-CD90+ (n = 21 for Case 1, n = 16 for Case 2), CD34+CD38+ (n = 26 for Case 1, n = 25 for Case 2) and CD34-CD33+ fractions (n = 11 for both cases) and each gene was evaluated by qRT-PCR. In the plots of melting curves (horizontal axis; temperature (°C), longitudinal axis; fluorescence), primer-specific curves were drawn in blue, curves derived from non-specific amplicons in red and a curve for negative control in light blue. Samples with non-specific melting curves were excluded for *EVI1* expression analysis. (a) C_t_ values of *beta-actin* were shown and C_t_ = 20 was set as a threshold value of *beta-actin* positivity (left). Melting curves of the samples were also shown (right). (b) C_t_ values of *gapdh* were shown and C_t_ = 20 was set as a threshold value of *gapdh* positivity (left). Melting curves of the samples were also shown (right). (c) C_t_ values of *BCR-ABL* were shown on the left and melting curves of the samples were shown on the right. Samples positive for *BCR-ABL* were identified as those derived from CML-CP cells. (d) After qRT-PCR analysis of the samples as to *beta-actin*, *gapdh* and *BCR-ABL*, several samples were excluded from the single cell analysis of *EVI1*. The number of samples for *EVI1* expression analysis turned to be 21 CD34+CD38-CD90+ cells, 24 CD34+CD38+ cells and 11 CD34-CD33+ cells for Case1, and 12 CD34+CD38-CD90+ cells, 25 CD34+CD38+ cells and 11 CD34-CD33+ cells for Case 2. Melting curves of the samples for human *EVI1* were shown.

**Supplementary Figure S2.**

(a) FCM gating strategy for the identification of GFP (Evi1)-high population from BM of Evi1-reporter CML model mice. Histograms of GFP from retroviral CML-CP mice (left) and transgenic CML mice (right) were shown. *Evi1^+/+^* allele mice were used as ‘fluorescence minus one’ control to set GFP-high gate. (b) Representative FCM data of BM cells from Evi1-reporter CML-CP mice. In BCR-ABL (KuOr)-positive BM cells, almost Evi1-high cells were Gr-1- while Evi1-low cells were mostly Gr-1+. In Lin- cells, Evi1-high cells had predominantly LSK immunophenotype in contrast to Evi1-low ones. (c) Representative FCM data of spleen cells from Evi1-reporter CML-CP mouse. When BCR-ABL (KuOr)-positive spleen cells were analyzed with lineage markers, Sca-1 and c-kit, LSK population showed the highest GFP intensity. (d) GFP-positive rates of stem/progenitor fractions and whole cells in the spleen of Evi1-reporter CML-CP mouse were shown. LSK cells had the highest GFP expression (n = 5). (e) 500 Evi1-high or -low CML-CP LSK cells were sorted and cultured on OP-9 stromal cell layer for seven days. Representative images of cultured LSK cells were shown (n = 3). Scale bar shows 100 μm.

Data are mean ± SD. *p < 0.01

**Supplementary Figure S3.**

(a) Kaplan-Meier plot of the survival of *Evi1^+/GFP^ BCR-ABL^tg/-^* mice (n = 30). (b) HE staining of BM from *Evi1^+/GFP^ BCR-ABL^-/-^* mice (Normal) or *Evi1^+/GFP^ BCR-ABL^tg/-^* mice (CML). (c) Vehicle or nilotinib (75 mg/kg daily) were administered orally to *Evi1^+/GFP^ BCR-ABL^tg/-^* mice for a week just after the CML development and the number of residual BM cells in femur (left) and the weight of spleen (middle) were calculated (Normal; with vehicle, CML; with vehicle, CML nilo; with nilotinib, n = 6 mice per group). The number of LSK cells also increased in CML mice (right, Normal; n = 4, CML; n = 6). (d) Representative FCM data of BM cells from *Evi1^+/GFP^ BCR-ABL^tg/-^*mouse. Expression profiles of lineage marker in Evi1-high (upper) or -low (lower) *Evi1^+/GFP^ BCR-ABL^tg/-^*BM cells are shown (left six panels). Expression levels of c-kit and Sca-1 were examined in *Evi1^+/GFP^ BCR-ABL^tg/-^*CML Lin- cells (right two panels). The mean percentage of each quadrant or area is shown. (e) Semisolid colony assay was done with 1000 Evi1-high or Evi1-low *Evi1^+/GFP^ BCR-ABL^tg/-^*CML LSK cells (n = 7). (f) Donor chimerism for multilineage (Myeloid; Gr-1, B lymphoid; B220, T lymphoid; CD4/CD8) reconstitution of Evi1-high or -low *Evi1^+/GFP^ BCR-ABL^tg/-^*CML LSK-transplants at 16 weeks post transplantation (n = 8 mice per group). (g) Evi1-high or Evi1-low *Evi1^+/GFP^ BCR-ABL^tg/-^*CML LSK cells were cultured in RPMI1640 medium (supplemented with 50 ng/mL SCF, 50 ng/mL TPO, 10 ng/mL IL-6) for 48 h with DMSO, 1 μM nilotinib or 5 μM nilotinib followed by semisolid colony assay (upper). The inhibitory rate (lower) in colony formation by nilotinib was calculated as follows: (Colony number in nilotinib-treated cells) / (Colony number in DMSO-treated cells) (n = 6 per group) (h) Representative FCM data of BM and spleen cells from nilotinib-treated *Evi1^+/GFP^ BCR-ABL^tg/-^*mouse (left). The summary of proportions of CD150+CD48- (long-term HSC; LT), CD150+CD48+ (short-term HSC; ST), CD150-CD48+ (multipotent progenitor; MPP) and CD150-CD48- (double negative; DN) fractions in LSK cells was shown (right; n = 5 per group). The differences between the proportions of LT in LSK cells were statistically evaluated.

Data are mean ± SD. ^+^p < 0.05, *p < 0.01, **p < 0.001, ***p < 0.0001, n.s.; not significant

**Supplementary Figure S4.**

(a) Images of colonies derived from Evi1-high (upper) or -low (lower) CML-BC LK cells. (b) *HOXA9* mRNA expression of each population from CML-BC mouse was measured by qRT-PCR (n = 3). (c) Evi1-high or Evi1-low CML-BC LK cells were cultured in RPMI1640 medium (supplemented with 50 ng/mL SCF, 50 ng/mL TPO, 10 ng/mL IL-6) for 48 h with DMSO, 1 μM nilotinib or 5 μM nilotinib followed by semisolid colony assay (left). The inhibitory rate (right) in colony formation by nilotinib was calculated as follows: (Colony number in nilotinib-treated cells) / (Colony number in DMSO-treated cells) (n = 6 per group) (d) Vehicle or nilotinib (75 mg/kg daily) were administered orally to Evi1-reporter CML-BC mice for a week from day 10 post BMT and the weight of spleen (left) and liver (right) were examined (n = 11). (e) Kaplan-Meier plot of the survival of sublethally irradiated recipient mice receiving Evi1-high or Evi1-low CML-BC LK cells from nilotinib-treated CML-BC mice (n = 3, p = 0.025).

Data are mean ± SD. *p < 0.01, **p < 0.001

**Supplementary Figure S5.**

(a) Experimental design of Evi1-reporter AML mice. 5FU-primed *Evi1^+/GFP^* BM cells with retroviral MLL-ENL, MOZ-TIF2 or TEL-PDGFR + AML1-ETO (TP+AE) were transplanted into lethally irradiated recipient mice. (b) Dot plots of GFP-positive rates of BM cells in Evi1-reporter AML mice, CML-BC mice and CML-CP mice were shown (n = 13 for CML-BC mice, n = 11 for MLL-ENL AML mice, n = 5 for MOZ-TIF2 AML mice, n = 5 for TP+AE AML mice and n = 5 for CML-CP mice). (c) Semisolid colony assay was done with 5000 Evi1-high or -low MLL-ENL AML cells (n = 7 for Evi1-high cells, n = 9 for Evi1-low cells). (d) Kaplan-Meier plot of the survival of primary recipient mice transplanted with MLL-ENL overexpressing cells (n = 12) and secondary recipient mice transplanted with 1000 or 100 c-kit+ Evi1-high or –low MLL-ENL overexpressing cells (n = 14 for 1000 c-kit+ Evi1-high, n = 17 for 1000 c-kit+ Evi1-low and n = 14 for 100 c-kit+ Evi1-high or –low MLL-ENL cells).

Data are mean ± SD. *p < 0.01, **p < 0.001, n.s.; not significant
